# Bimanual Examination of the Bladder

**Published:** 1896-11-21

**Authors:** 


### BIMANUAL EXAMINATION OF THE BLADDER.

We are all accustomed to the bimanual method of examination in gynæcological practice. Probably many do not so constantly bear in mind its advantages in the investigation of diseases of the bladder. By placing the index finger of one hand in the rectum or vagina, and pressing firmly with the other hand on the abdominal walls immediately above the pubes, the bladder being empty, results of considerable value may sometimes be obtained. Mr. Henry Morris, writing in the *Lancet*, shows that this method of examination is especially useful in the investigation of cases of hæmaturia, in which it may be difficult or impossible to keep the bladder sufficiently free from blood to obtain a clear view by the cystoscope. In cases of vesical calculus, in which the stone falls behind the prostate or is encysted, in papillomata, and in some cases of malignant ulceration of the bladder, bimanual examination may give information which it is difficult to obtain by any other simple method, and by making clear the diagnosis in doubtful cases of hæmaturia, may often be the means of preventing unnecessary exploration of the kidney.

